# Why *Plasmodium vivax* and *Plasmodium falciparum* are so different? A tale of two clades and their species diversities

**DOI:** 10.1186/s12936-022-04130-9

**Published:** 2022-05-03

**Authors:** Ananias A. Escalante, Axl S. Cepeda, M. Andreína Pacheco

**Affiliations:** Biology Department/Institute of Genomics and Evolutionary Medicine [iGEM], Temple University, Philadelphia, PA 19122-1801 USA

**Keywords:** Chitinases, *Laverania*, Malaria, Merozoite surface protein, Molecular clock, Phylogenomics, *Plasmodium* phylogeny, *PfRH5-PfCyRPA-PfRipr* complex

## Abstract

The global malaria burden sometimes obscures that the genus *Plasmodium* comprises diverse clades with lineages that independently gave origin to the extant human parasites. Indeed, the differences between the human malaria parasites were highlighted in the classical taxonomy by dividing them into two subgenera, the subgenus *Plasmodium*, which included all the human parasites but *Plasmodium falciparum* that was placed in its separate subgenus, *Laverania*. Here, the evolution of *Plasmodium* in primates will be discussed in terms of their species diversity and some of their distinct phenotypes, putative molecular adaptations, and host–parasite biocenosis. Thus, in addition to a current phylogeny using genome-level data, some specific molecular features will be discussed as examples of how these parasites have diverged. The two subgenera of malaria parasites found in primates, *Plasmodium* and *Laverania*, reflect extant monophyletic groups that originated in Africa. However, the subgenus *Plasmodium* involves species in Southeast Asia that were likely the result of adaptive radiation. Such events led to the *Plasmodium vivax* lineage. Although the *Laverania* species, including *P. falciparum*, has been considered to share “avian characteristics,” molecular traits that were likely in the common ancestor of primate and avian parasites are sometimes kept in the *Plasmodium* subgenus while being lost in *Laverania*. Assessing how molecular traits in the primate malaria clades originated is a fundamental science problem that will likely provide new targets for interventions. However, given that the genus *Plasmodium* is paraphyletic (some descendant groups are in other genera), understanding the evolution of malaria parasites will benefit from studying “non-*Plasmodium*” Haemosporida.

## Background

*Plasmodium* species infecting humans are part of different evolutionary lineages or clades; those lineages independently gave rise to human parasites that shared recent common ancestors with other species in nonhuman primates [1, 2]. Not surprisingly, the five parasites that primarily cause malaria in humans show biological differences in almost any stage of their life cycles [3, 4].

For example, a critical process in how *Plasmodium* species infect humans is the invasion of red blood cells. While *Plasmodium falciparum* does not show tropism toward a specific red blood cell age, *Plasmodium vivax* and the two species under *Plasmodium ovale *sensu lato (s.l.) (*P. o. curtisi* and *P. o. wallikeri*) invade young red blood cells or reticulocytes. In contrast, *Plasmodium malariae* has been proposed to invade old red blood cells [3, 5–7]. There are also differences in the time of gametocyte production and their lifespan, which are fundamental fitness components [8, 9]. *Plasmodium falciparum* undergoes five stages of development in 9–12 days, the longest maturation time, and remains infectious for several days compared with other malaria parasites. On the other hand, *Plasmodium vivax* and the *P. ovale* s.l. develop dormant stages or hypnozoites that cause relapse after the primary infection; such stages are not found in *P. falciparum* or *P. malariae* [3, 10]. Another example is that *P. falciparum*-infected erythrocytes can adhere to the endothelium of capillaries and venules [11, 12]. This process known as sequestration is linked to severe clinical presentations that are infrequent in non-*falciparum* malaria [13, 14]. The traits listed above are a few out of many differences among the *Plasmodium* causing malaria in humans.

In addition to *Plasmodium* infecting humans as their primary host, there are zoonotic malaria parasites from nonhuman primates. The most recognizable is *Plasmodium knowlesi* from macaques in Southeast Asia [15–17]. However, evidence incriminates other nonhuman primate malaria species as zoonoses in that region, even causing asymptomatic infections [17–19]. Furthermore, anthropozoonotic malaria parasite cycles involving South American nonhuman primates have been reported [20–22].

The parasite species that can infect humans vary in their geographic distributions and ecological contexts. There could be a predominant species in a region or all of them [1, 23, 24]. In contrast, zoonotic and anthropozoonotic cycles have restricted geographic distributions limited by biogeographical factors involving the presence of nonhuman primates and suitable vectors [1, 20, 22]. Thus, the local *Plasmodium* species pool in primates (human and nonhuman) complicates malaria epidemiology to the extent of hampering elimination efforts [15–17, 20–22].

Although zoonotic species have been the focus of attention, there are at least 39 known *Plasmodium* in nonhuman primates worldwide; that number includes described species or detected lineages (Table 1). Several of these parasites were not a particular focus of research inquiry until recently. It can be speculated that such limited attention was due to difficulties working in nonhuman primates. Also, there was a reasonable focus on workable animal models to understand malaria biology and explore treatments or vaccines. Regardless of the factors that hindered their study, extraordinary progress has been made in the last two decades.

**Table 1:** List of primate malarias and the avian *Plasmodium* used in comparative studies

Haemosporidian species	Natural host	Biogeographic region	Refs.
*Plasmodium (Haemamoeba) gallinaceum*	Galliformes	Oriental zoogeographical region	[45]
*Plasmodium (Haemamoeba) relictum*	More than 300 bird species	Worldwide distribution	[45]
***Plasmodium (Laverania) falciparum***	***Homo sapiens***	**Worldwide Tropical regions**	[3]
*Plasmodium (Laverania) praefalciparum*	*Gorilla gorilla*	African region (e.g., Cameroon)	[2, 73]
*Plasmodium (Laverania) blacklocki*	*Gorilla gorilla*	African region (Cameroon, Uganda)	[2, 73]
*Plasmodium (Laverania) adleri*	*Gorilla gorilla*	African region (Cameroon)	[2, 73]
*Plasmodium (Laverania) reichenowi*	*Pan troglodytes*	African region (e.g., Congo)	[3, 73]
*Plasmodium (Laverania) billcollinsi*	*Pan troglodytes*	African region (e.g., Uganda, Republic of the Congo)	[42, 73]
*Plasmodium (Laverania) gaboni*	*Pan troglodytes*	African region (Cameroon, Democratic Republic of Congo, Gabon, Kenya, Republic of Congo, Uganda)	[44, 73, 85]
*Plasmodium (Laverania) billbrayi*	*Pan troglodytes*	African region (Uganda, Republic of the Congo)	[42, 73]
*Plasmodium (Laverania) lomamiensis*	*Pan paniscus*	African region (Democratic Republic of Congo)	[43]
***Plasmodium (Plasmodium) malariae***	***Homo sapiens, Pan troglodytes***	**Worldwide**	[3]
*Plasmodium (Plasmodium) malariae*-like	*Pan troglodytes*	African region (e.g., Gabon)	[3, 81]
*Plasmodium (Plasmodium) brasilianum*	Haplorhini, Simiiformes (*Alouatta* spp., *Brachyteles* sp., *Cacajao* sp.*, Callicebus* sp. *Lagothrix* sp.*,* sp.*, Mico* sp*., Pithecia* sp.*, Saguinus* sp.*, Saimiri* sp.)	South America	[3, 81]
***Plasmodium (Plasmodium) ovale curtisi***	***Homo sapiens, Pan troglodytes***	**Worldwide**	[81]
***Plasmodium (Plasmodium) ovale wallikeri***	***Homo sapiens, Pan troglodytes***	**Worldwide**	[81]
*Plasmodium (Plasmodium) gonderi*	*Cercocebus agilis, C. atys, Lophocebus albigena, Mandrillus leucophaeus, M. sphinx*	African region	[3]
*Plasmodium (Plasmodium)* sp. (DAJ-2004)	*Cercopithecus cephus, C. nictitans, Mandrillus sphinx*	African region	[36]
*Plasmodium (Plasmodium) hylobati*	*Hylobates moloch, H. muelleri*	Asian region (e.g., West Malaysia)	[3]
*Plasmodium (Plasmodium) eylesi*	*Hylobates lar*	Asian region (e.g., Indonesia, Malaysia)	[3]
*Plasmodium (Plasmodium) youngi*	*Hylobates lar*	Asian region (e.g., Malaysia)	[3]
*Plasmodium (Plasmodium) jefferyi*	*Hylobates lar*	Asian region (e.g., Malaysia)	[3]
*Plasmodium (Plasmodium) fragile*	*M. radiata, M. mulatta, Prebytis* spp.	Asian region (e.g., Southern India, Sri Lanka)	[3, 50]
*Plasmodium (Plasmodium) fieldi*	*Macaca arctoides, M. fascicularis, M. leonina, M. nemestrina*	Asian region (e.g., Sri Lanka)	[3, 50]
*Plasmodium (Plasmodium) simiovale*	*Macaca sinica*	Asian region (e.g., West Malaysia, Thailand)	[3, 50]
*Plasmodium (Plasmodium) inui*	*Macaca arctoides, M. cyclopis, M. fascicularis, M. leonina, M. mulatta, M. nemestrina, M. nigra, M. radiata, M. sinica, Trachypithecus cristatus, T. obscurus*	Asian region (Bangladesh, Borneo, Cebu, Southwest India, Java, West Malaysia, Mindanao, Nicobar Islands, Sri Lanka, Palawan, Sulawesi, Taiwan, Thailand, Tioman, Vietnam	[3, 19, 45, 50–55, 76]
*Plasmodium (Plasmodium) knowlesi*	*Macaca arctoides, M. fascicularis, M. nemestrina, Presbytis femoralis, Trachypithecus obscurus,*	Asian region (Southeast)	[3, 19, 45, 50–55, 76]
*Plasmodium (Plasmodium) coatneyi*	*Macaca arctoides, M. fascicularis, M. nemestrina, Trachypithecus cristatus*	Asian region (Malaysia, Philippines, Cebu, Kalimantan, Palawan, Sarawak, Thailand)	[3, 19, 45, 50–55, 76]
*Plasmodium (Plasmodium) cynomolgi*	*Macaca arctoides, M. fascicularis, M. leonina, M. mulatta, M. nemestrina, M. radiata, M. sinica, Semnopithecus priam, Trachypithecus cristatus*	Asian region (Bangladesh, Borneo, Cambodia, Cebu, Southwest India, Java, West Malaysia, Nicobar Islands, Palawan, Sri Lanka, Taiwan, Thailand)	[3, 19, 45, 50–55, 76]
*Plasmodium (Plasmodium) pitheci*	*Pongo pygmaeus*	Asian region (Borneo)	[3, 30, 56]
*Plasmodium (Plasmodium) silvaticum*	*Pongo pygmaeus*	Asian region (Borneo)	[3, 30, 56]
*Plasmodium (Plasmodium) schwrtzi*	*Pan troglodytes, Gorilla gorilla*	African region (Cameroon)	[3]
*Plasmodium (Plasmodium) simium*	*Alouatta caraya, A. guariba, Brachyteles arachnoides*	South America (Brazil-Atlantic Forest)	[3, 20, 21]
*Plasmodium vivax-like*	*Pan troglodytes, Gorilla gorilla*	African region	[48]
***Plasmodium (Plasmodium) vivax***	***Homo sapiens***	**Worldwide Tropical regions**	[3]
*Plasmodium girardi*	*Lemur fulvus rufus*	Madagascar	[3]
*Plasmodium lemuris*	*Lemur collaris*	Madagascar	[3]
*Plasmodium percygarnhami*	*Eulemur macaco macaco*	Madagascar	[25, 60]
*Plasmodium foleyi*	*Eulemur rufus*	Madagascar	[60]
*Plasmodium coulangesi*	*Eulemur macaco*	Madagascar	[60]
*Plasmodium bucki*	*Eulemur macaco*	Madagascar	[60]
*Plasmodium uilenbergi*	*Eulemur fulvus*	Madagascar	[60]
*Plasmodium malagasi*	*Propithecus verreauxi*	Madagascar	[60]
*Plasmodium* sp. (haplotype A)	*Hapalemur griseus griseus*	Madagascar	[25]
*Plasmodium* sp. (haplotype B)	*Varecia variegata*	Madagascar	[25]
*Plasmodium* sp. (haplotype C)	*Hapalemur griseus griseus*	Madagascar	[25]
*Plasmodium* sp. (haplotype D)	*Indri indri*	Madagascar	[25]
*Plasmodium* sp. (haplotype E)	*Eulemur macaco*	Madagascar	[25]

The parasite species that infect humans as their primary host are in bold. Those species that are part of zoonotic or anthropozoonotic (reverse zoonoses) cycles are underlined

This review focuses on the evolution of *Plasmodium* in primates. Studying how distinct phenotypes, molecular adaptations, and host–parasite biocenosis emerged during the evolutionary history of *Plasmodium* in primates may lead to new intervention targets and a better understanding of host-parasite interactions. Indeed, there is much to be gained from comparative approaches that include nonhuman primate malaria parasites. For example, antigenic variation was first discovered in *P. knowlesi* [26, 27], a convergent trait found in *P. falciparum* later [14, 28, 29].

Here, *Plasmodium* diversity in primates will be considered at different levels. First, an overview of the primate malarias species diversity will be provided by following a phylogeny. Then a discussion on what is known about their host specificity will be followed by examples of how comparative genomics allow detecting putative molecular adaptations or functional differences. Finally, a discussion of the timeframes estimated for these parasites’ divergence will be provided.

### *Plasmodium* in primates: an overview

*Plasmodium* species in nonhuman primates were first found in orang-utans and macaques imported to Europe between 1905 and 1907 [3, 4, 30]. A decade later, malaria parasites in African Apes were found by Eduard Reichenow [31, 32]. Unlike those parasites from Southeast Asia, the early findings in gorillas and chimpanzees were considered infections of the species found in humans [3, 33–35]. However, it was soon established that those were distinct species [33, 34].

As nonhuman primate malarias were discovered, parallels were established with those found in humans [4]. This search for similarities between nonhuman primate parasites and human parasites (phenetic approach) is evidenced in the book “The primate malarias,” organized by “malaria’s types” rather than by the parasites’ inferred evolutionary relationships or proposed taxonomy [3]. Nowadays, it has been demonstrated that these phenotypes are convergent traits among *Plasmodium* species [36–40], so they do not inform about the parasites’ evolutionary history. A notorious example *P. malariae* and *Plasmodium inui*, the latest species found in macaques (Table 1)*;* they both have a 72-h periodicity (or quartan malaria) but were not considered “closely related” or sister taxa [4, 36, 37]. Nevertheless, such comparisons between human and nonhuman primate parasites were pragmatic in establishing putative models and understanding their potential disease risk to humans, acknowledging the lack of a robust evolutionary framework [3, 4].

**Fig. 1:**
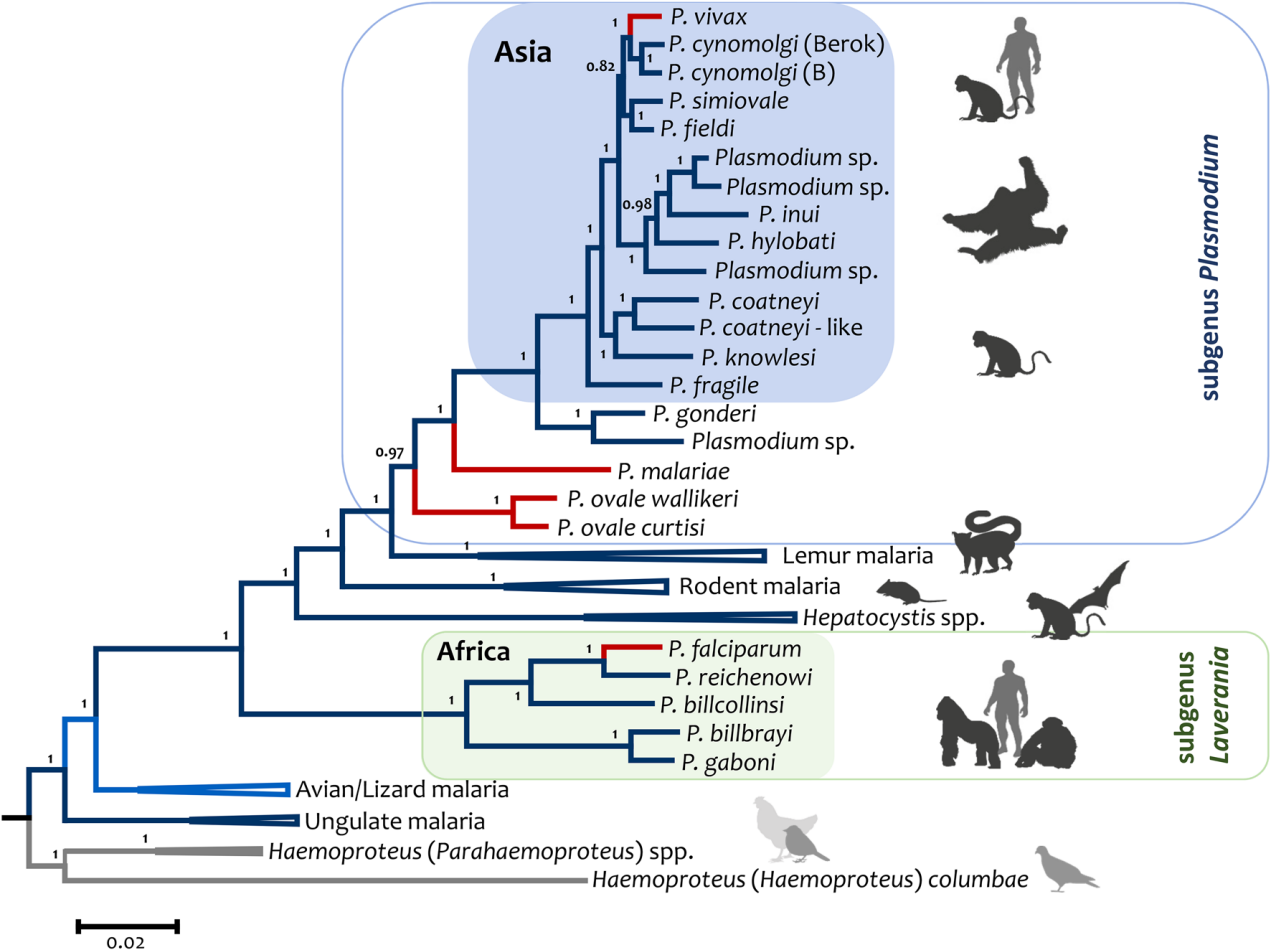
Phylogenetic tree of *Plasmodium* spp. based on complete mitochondrial genomes. Bayesian and Maximum Likelihood methods yielded identical topologies; only the Bayesian tree obtained using MrBayes v3.1.2 is shown. The alignment included approximately 5800 bp of the parasites’ mitochondrial genomes (mtDNA). The values above branches are posterior probabilities. The phylogeny branches leading to human malaria parasites are colored in red

Here, *Plasmodium* species in primates will be discussed following their evolutionary history. The Bayesian phylogeny in Fig. 1 was estimated based on the mitochondrial (mtDNA) genome because it includes several known taxa in each clade. Although a single locus, the mtDNA genome has approximately the same AT content across *Plasmodium* species. Also, it is not saturated at the time scale of the events described in this phylogeny [39, 41]. Thus, these characteristics reduce the risk of model misspecification that could affect phylogenetic analyses. Importantly, the mtDNA phylogeny is concordant with the nuclear genes [38, 39], as will be discussed later.

Perhaps a faulty generalization, there are three notable species radiations of *Plasmodium* in primates that are known of yet. One occurred in great African apes comprising the lineages that gave origin to *P. falciparum*, the species causing the most severe form of malaria in humans. Such clade will be referred to as *Laverania* as it is a monophyletic group that coincides with the subgenus proposed in the classical malaria taxonomy (Table 1, Fig. 1) [4]. *Laverania* includes at least seven species found in African hominids other than humans (Table 1) [2, 42–44]. *Plasmodium falciparum* and related parasites have several unique genes and gene families that set it apart from the other *Plasmodium* in primates, which will be revised later.

Another important clade consists of parasites found in Africa and Southeast Asia (Table 1). This monophyletic group contains *Plasmodium* species found in Catharine’s primates in a complex evolutionary history involving the origin of *P. vivax* [39, 40, 45–47]. For lack of a better term, these parasites will be referred to as the “vivax clade” to link them with the origin of *P. vivax* (Fig. 1). Among those species are parasites found in orang-utans, gibbons, macaques, and langurs. It also includes lineages found in African apes that will be generically called *Plasmodium vivax-*like [47, 48]. Whether one of these “vivax*-*like*”* is the African ape parasite, *Plasmodium schwetzi* [3, 4], cannot be determined because there is no molecular data associated with that species.

Southeast Asia species belonging to the vivax-clade are part of rapid radiation involving multiple sympatric hosts in an area that has undergone complex biogeographic processes that includes the origin of *P. vivax* [40, 45, 48]. A unique characteristic of this monophyletic group is that it has species with different life-history traits. In particular, there is a quartan parasite (72 h in *P. inui* a convergent trait with *P. malariae*), a quotidian (24 h cycle for *P. knowlesi,* unique in primates but found in avian and rodent malarias), and tertian malarias (48 h cycle common in all the other primate *Plasmodium*) [3]. The species differ in their tropisms toward types of red blood cells. *Plasmodium cynomolgi* and *Plasmodium coatneyi* invade reticulocytes*,* whereas *P. knowlesi* erythrocytes of all ages [3, 26]. *Plasmodium knowlesi* has the *SICAvar* gene family associated with antigenic variation, an analog (convergent trait) to the *var* gene family in *Laverania* [49]. On the other hand, *P. cynomolgi*, *Plasmodium simiovale,* and *Plasmodium fieldi* relapse like *P. vivax*; whereas *P. knowlesi*, *P. coatneyi,* and *Plasmodium fragile*, do not [10].

It can be hypothesized that such phenotypic diversity may allow the coexistence of multiple parasite species by reducing competition [45]. Indeed, coinfections are relatively common in macaques (individuals with more than one species of *Plasmodium*), and these species are all transmitted by the same vectors in each region [45, 50–55]. In that regard, it is similar to humans, where malaria parasites coinfections are relatively common but, in the human case, by species with different evolutionary histories rather than species from the same monophyletic group. Thus, considering that these rapid speciation events were linked to divergent phenotypes, it suggests adaptive radiation [45]. It is worth noting that there is no molecular information available from three species of *Plasmodium* described in gibbons, *Plasmodium eylesi*, *Plasmodium jefferyi*, and *Plasmodium youngi* [3]*.* It is assumed that these parasites are part of the same vivax-clade in Southeast Asia, but data from those species could change this perspective.

There are two parasites described in orang-utans that are likely part of the *Plasmodium* species’ radiation in southeast Asia, *Plasmodium pitheci* and *Plasmodium silvaticum* [30, 56]. They are tertian malarias like *P. vivax*. Although no molecular information is linked to these two species, three *Plasmodium* lineages from orang-utans have been reported using mtDNA (Fig. 1) and other nuclear loci. Whether those three molecular lineages include the two described species requires additional information. Nevertheless, based on the molecular evidence, the orang-utan parasites with molecular evidence are part of a clade with *P. inui* (Fig. 1), a quartan parasite commonly found in macaques, together with *Plasmodium hylobati*, a tertian parasite from gibbons [45, 46]. There are malaria parasites in African monkeys that are also part of the “vivax-clade” (Table 1). In particular, *Plasmodium gonderi* and another lineage that could be *Plasmodium petersi* (Fig. 1), but the latest has not been confirmed [57]. *Plasmodium gonderi* is a tertian parasite with tropism toward reticulocytes, like others in the “vivax-clade.”

Not considered part of the “vivax” or Laverania clades, there are two lineages involving sisters’ taxa of the human parasites *P. malariae* and *P. ovale* s.l. [2, 42, 58], the latest harbours two separated cryptic species, *P. o. curtisi* and *P. o. wallikeri* [58, 59]. However, it is worth mentioning that the two *P. ovale* s.l. species, *P. malariae*, and the vivax-clade are part of a monophyletic group corresponding with the subgenus *Plasmodium* [25, 38, 39] (Fig. 1).

Finally, the third radiation of malaria parasites is a less-known clade of *Plasmodium* in lemurs that includes several putative species [25, 60]. Unfortunately, the eight described morphospecies are controversial and lack molecular data (Table 1) [25, 60]. How these parasites in lemurs radiated, their diversity, and their relationship with the continental nonhuman primate malarias are neglected issues in the research agenda on the evolution of *Plasmodium*. Although initially included in the subgenera *Vinckeia* with the rodent malarias [4], these lemur parasites may share a common ancestor with the non-Laverania primate malarias based on mtDNA (Fig. 1) [25]. If confirmed, the lemur malaria clade will also be part of a broader monophyletic group comprised of all non-Laverania primate malarias*.*

### *Plasmodium* in primates: host specificity

As in other parasites, there is compelling evidence indicating host switches among primate malaria parasites through their evolutionary history [40, 42, 45, 61–64]. Host switches have fueled questions regarding where nonhuman parasites change disease risk to humans. This concern is not new [3, 65]. However, its importance was evidenced by discovering naturally infected humans with *P. knowlesi* in high prevalence [15]. A different problem is discussing how host specificity, or lack of it, may have driven the diversity of primate malaria parasites. Indeed, studying parasite speciation or the parasites' actual host range is different from assessing the prevalence of zoonotic infections. For example, a parasite can fail in colonizing humans because there is no human-to-human transmission. However, it could still be relevant because of its impact on disease as a zoonosis.

Classic parasite speciation models are a gradient between two processes, codivergence and host switches [66–68]. Codivergence involves that parasites are shared by descent while their hosts diverge because of determined biological traits [66, 69]. On the other hand, host switches imply that new malaria parasites host biocenoses change due to their ecological contexts, leading to the subsequent specialization to a new host [45, 70, 71]. In the latter scenario, geographical proximity across their hosts’ evolutionary histories is crucial to allow for opportunities for parasite transfers [45, 67, 70, 72]. This second scenario gains support wherever there are no phylogenetic concordances between host and parasite species. Considering such a framework, *Plasmodium* in primates involves a series of biogeographic processes where the hosts’ demographic histories limit the possibility of host-switches regionally. At the same time, there is some level of host specificity comprising specific clades of primates.

There is not a clade of gorilla versus a clade of chimpanzee parasites; thus, in that regard, cospeciation did not occur. However, there are pairs of parasites between chimpanzee and gorilla lineages in the Laverania clade that could indicate cospeciation. In particular, *Plasmodium gaboni* (chimpanzee) and *Plasmodium adleri* (gorilla) are sister taxa, and then *P. reichenowi* (chimpanzee) and *Plasmodium praefalciparum* (gorilla) are also sister taxa [73]. Unfortunately, the genomic data is still incomplete due to the absence of the bonobo parasite *Plasmodium lomamiensis.* However, the bonobo parasite seems to be a sister taxon of *P. reichenowi* found in chimpanzees [2, 43]*.* The divergence of bonobo and chimpanzee parasites may suggest cospeciation. Disentangling such paleobiogeographic scenarios requires additional information*.*

Interestingly, there is a vivax-like parasite in African apes, but no evidence indicates it can infect humans [48]. Thus, it seems that colonizing nonhuman African apes has led to new and divergent vivax-like lineages [48]. So, in this case, a host switch likely originated two different species of *P. vivax-*like parasites [48]. An interesting case is *Plasmodium simium,* as it seems to be differentiating from *P. vivax* populations [21]. Whether this is a case of early speciation remains to be elucidated since *P. simium* infects humans [20], which may facilitate introgression.

The situation is far more complex in Southeast Asia within the species in the vivax-clade. The parasites commonly referred to as “macaque malarias” [50] vary in their host specificity (Table 1). Although humans are infected, humans seem to be paratenic hosts since human-to-human transmission has not been documented [74, 75]. There is no evidence of host-switches between “macaque” and orang-utans parasites, regardless of their hosts’ overlapping distributions [45, 54, 76]. This observation is significant considering that all the species incriminated as vectors of nonhuman primate *Plasmodium* belong to the *Anopheles* (*Leucosphyrus*) group, and they feed on macaques and orang-utans [56, 75]. There is a lack of sampling effort in other primates, so it is premature to disregard those species as potential hosts for the so-called macaque malarias.

Nevertheless, the limited host range observed in some species (e.g., orang-utan malaria parasites) suggests patterns of concordance between parasites and particular clades of primates. Out of Southeast Asia, for example, the tertian *Laverania* parasites radiated and are transmitted within Homininae (*Homo*, *Pan*, *Gorilla*). Host specialization in malaria parasites can be selected for in some circumstances, as the data in humans and other apes suggest. For example, it can be hypothesized that following the human expansion, molecular adaptations associated with the erythrocyte invasion in *P. falciparum*, such as *Pf*EBA165 and *Pf*RH5, were selected for narrowing its host range to humans [77, 78]. Furthermore, there is no evidence of nonhuman great apes parasites infecting humans, even in close contact with infected individuals [73, 79, 80]. Likewise, the tertian species in Asian apes seem to be host-specific (*Pongo*, *Hylobates*), whereas *P. ovale* s.l. species are restricted to African apes (Homininae) [59, 81]. All these patterns indicate that the host ranges of primate malaria parasites likely underwent historical changes due to each host species’ demographic history, driving their population densities and spatial distributions [45]. Thus, in some contexts, specialist *Plasmodium* parasites may have been selected for when their hosts' populations were expanding, as seems to be the case of *P. falciparum*, *P. vivax*, and other parasites*.*

The case of *P. malariae/Plasmodium brasilianum* species deserves particular attention. This parasite shows host restriction in Africa by infecting African apes (Homininae), coinciding with the geographic region where *P. malariae* very likely originated [2, 42, 81]. Furthermore, there is no evidence that apes in Asia or other nonhuman primates acquire *P. malariae* locally. Thus, this parasite has a limited host range in the old world among Homininae. The implication is that *P. malariae* became a generalist (*P. brasilianum*) after its introduction in the New World by infecting multiple nonhuman primate species across many genera in all families of local primates.

Disease ecology’s available theory predicts the traits that introduced parasites may have to establish in a new locality. First, such parasites are generalists; second, they likely have low virulence in the natural host and should be prevalent enough to be present in the host population that introduced them into the new environment. Third, there should be suitable competent vectors in the new area. Overall, these dynamics are affected by the endemic parasite assemblage [82, 83]. Thus, it can be hypothesized that *P. malariae* became generalist in the New World facilitated by vectors with a broad host range, low virulence in the natural host (humans), and no endemic parasite that could compete with them.

It is worth noting that spillovers from humans to nonhuman primates are being documented in Asia and Africa. *Plasmodium falciparum* has been found in gorillas and chimpanzees in captivity or semi-captivity settings [73, 84, 85]. In one of those studies, the parasites were positive for chloroquine resistance mutations [85]. Likewise, there are reports of *P. malariae* in chimpanzees [42, 86]. The two *P. ovale* sub-species circulate in chimpanzees from Cameroon, low land gorillas from the Central African Republic, and bonobos [43, 64, 84, 87]. Whether this could lead to anthroponotic cycles (reverse zoonosis) is premature to say.

### Estimating the phylogeny of *Plasmodium* in primates: from single gene trees to phylogenomics

Molecular phylogenetic analysis in malarial parasites started in the early 1990s. Like in other eukaryotic protozoa, the original locus of choice was the 18s SSU rRNA, and those studies inquired about the origins of human malarias [88–91]. Although the 18s SSU rRNA is still widely used in diagnostics, its use in phylogenetic studies has diminished within Haemosporida. In particular, the occurrence of non-concerted evolution among stage-specific expressed paralogs makes interpreting gene trees in terms of species trees challenging [92, 93].

Other early phylogenetic studies used the gene encoding the circumsporozoite protein (CSP) [94]. The CSP is an antigen expressed on the surface of the sporozoite, the infective stage inoculated by the vector into the vertebrate host. It has been a target of pre-erythrocytic vaccines because of its critical functions in the first steps of the parasite infection. However, it is under selective pressure for accumulating polymorphism with a tandem repeat motif (low complexity region) that does not allow its full-length alignment [94]. Such characteristics are not suitable for inferring species trees. Still, it has been appropriately used in some contexts considering the extensive database of sequences available [15].

Finally, the parasite’s mitochondrial cytochrome b gene (*cytb*) was used in a haemosporidian phylogeny [36] to overcome the shortcomings of the two first loci. Overall, it corroborated early observations. A commonality in such early studies (*csp*, *cytb*, and 18S SSU rRNA) was that they aimed to infer the evolutionary history of known taxa. Perhaps their more significant conclusion was that the human malarias emerged independently. Nowadays, phylogenomic analyses can take advantage of the genomic data available.

A common problem when inferring the species tree from genome-level data is gene tree conflicts because of incomplete lineage sorting, differences in selection constraints, changes in the model of nucleotide substitution across loci, among other factors. As a result, there are multiple approaches to dealing with species trees versus gene trees. It is worth mentioning that the taxa limit phylogenomic analyses with the lower quality genome (e.g., shorter sequences or less genome coverage). It can only use single-copy genes and with clear orthologs across the species under consideration.

**Fig. 2:**
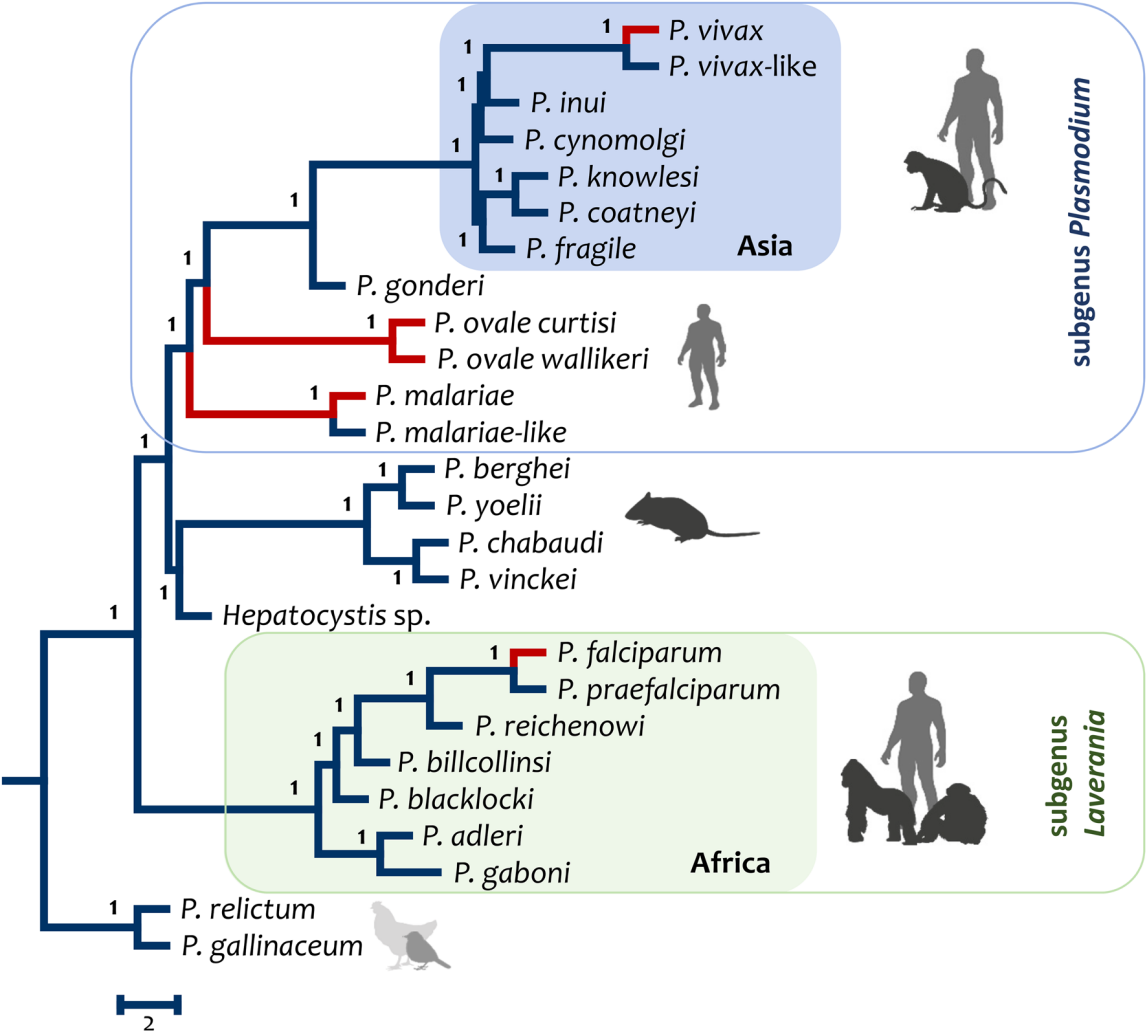
Phylogenomic analyses of the primate malarias using the available genomes. Consensus phylogeny on 1028 single-copy orthologous genes under the multi-species coalescent model implemented in ASTRAL III. *Plasmodium gallinaceum* and *Plasmodium relictum* were used as an outgroup to estimate the root of the primate malarias

Figure 2 shows a consensus phylogeny that incorporates 1028 single-copy genes; orthologous groups were inferred de novo by using OrthoFinder [95] on all available *Plasmodium* genomes from mammals and *Hepatocystis* [81, 96–99]*.* Two *Plasmodium* species from birds with genomic data, *Plasmodium gallinaceum* and *Plasmodium relictum*, were used to estimate the root of the primate-rodent malarias [100]. The species tree was inferred under the multi-species coalescent model implemented in ASTRAL III [101]; this method finds the species tree that agrees with the largest quartet trees from a set of gene trees. The gene trees were estimated using IQTREE [102] under the best substitution model that fit each gene alignment. It is worth mentioning that an identical topology was obtained by concatenating genes. Overall, the phylogeny recovers the major clades made evident by early studies and is consistent with the mtDNA phylogeny presented in Fig. 1, with a few differences that will be discussed below.

As expected, the *Laverania* subgenus relationships presented here (Fig. 2) are congruent with those previously reported [2, 97, 98]. It is a monophyletic group separated from the other *Plasmodium* found in mammals. *Plasmodium vivax-P. vivax-*like lineage (vivax lineage for short) is within the radiation of the parasites referred to as the Africa-Asian radiation, as previously proposed for *P. vivax* [38–40, 45, 48, 61, 98]. The primary difference is that the *vivax* lineage shares a more common ancestor with *P. inui* rather than *P. cynomolgi* [38–40, 46, 103].

*Plasmodium cynomolgi*, among the parasites found in Southeast Asia, has been widely accepted as the species that shared the most recent common ancestor with the vivax lineages [3, 39, 40, 91, 99]. This notion is supported by biological traits and early phylogenetic analyses using single-gene approaches (Fig. 1). Thus, the *P. inui*–*P. vivax* lineage relationship could result from limited sampling in terms of species with available genomes, particularly considering the fast cladogenesis of these species. Indeed, there is no genomic data from many species of parasites from gibbons or orang-utan. Those Asian ape parasites species share a common ancestor with *P. inui* based on the mitochondria genome data [45, 46]. Finally, it must be noted that preliminary data indicates great diversity within *P. cynomolgi* and *P. inui* [3, 45, 46]*.* Sampling across divergent “strains” would likely improve phylogenetic inferences about the relationship between the *P. vivax* lineage and parasites found in macaques.

The position of *P. gonderi* is also worth noticing. The phylogeny using genomic data (Fig. 2) concord with the mitochondrial phylogeny (Fig. 1) and several other studies [36, 38, 40, 62], but differs from other phylogenomic studies where the *vivax* lineage appears as a sister clade to the other macaque parasites [2]. Although the relative position of *Hepatocystis* is consistent with previous studies [36, 38, 98], its inclusion may have changed the relative position of *P. gonderi* in this analysis when compared to others [2]. This observation highlights the issue that the sampled taxa, not only loci, should be considered when comparing incongruences between phylogenetic studies. Finally, it is worth noting that there is at least one *Plasmodium* species found in mandrills that shares a common ancestor with *P. gonderi* (Fig. 1)*.* No genomic data is available from that parasite*.*

Based on the phylogenies presented in Figs. 1 and 2, it is reasonable to state that the phylogenies support an origin of *P. vivax* as part of the parasite radiation in Asia [40, 62]. Such observation will be consistent with an early introduction of the vivax-lineage from Southeast Asia into Africa, giving origin to the two species known in African apes, *P. vivax* in humans and *P. vivax-*like in chimpanzees [48]. Interestingly, in the phylogeny based on genome data presented here (Fig. 2), the subgenus *Plasmodium* is a monophyletic group, differing from the phylogenies estimated by others [2, 98]. The phylogeny also is consistent with *P. malariae* originating in Africa, as indicated by the presence of *P. malariae-*like [42, 81]. This parasite could be called *Plasmodium rhodaini*, if it ever re-described, to honor the old species name. A less explored clade is one where *P. ovale* and *P. malariae* share a common ancestor with all other primate malarias, including the poorly sampled parasites in lemurs [25]. Unfortunately, the genomic data is incomplete in terms of taxa.

### A few taxonomic notes

Whereas an issue discussed in taxonomy, delimiting parasite species has repercussions in health and policy [4, 104], e.g., differential diagnostics. The goal of a taxonomy is to integrate information and make biological predictions about the organisms considered part of particular taxa. Thus, discovering species and having them on record are aspects of critical importance [4].

*Plasmodium* species are described using life histories and morphological traits on the parasite blood stages observed in Giemsa-stained films in a light microscope. Unfortunately, even unrelated species can look alike, as evidenced by *P. knowlesi* in humans that can be confused with *P. falciparum* or *P. malariae* [105]. Nevertheless, there were enough traits to set species apart in parasites from Southeast Asia [3], those were reproduced in molecular studies. In contrast, all initial reports of parasites in African apes found them indistinguishable from *Plasmodium* in humans.

Thus, the host was used for species delimitation and identification in several cases [35]. Experimental infections were used wherever possible [3], but such a practice cannot be scaled up, and nowadays is ethically and scientifically questionable for taxonomic porpoises. However, after the parasite’s mitochondrial cytochrome b gene (*cytb*) was used in a haemosporidian phylogeny [36], molecular lineages started to be used as a proxy to discovering and delimiting species [42–44, 47, 106]. Decades after the original observation of *Plasmodium* in African apes, such mitochondrial loci allowed the discovery of distinct molecular lineages circulating among African apes that were independently reported and reproducible [2, 42–44, 47]. Such data indicated active transmission. Still, the argument was made against recognizing those *Plasmodium* species because there was a lack of certainty about their hosts. The sexual stages required to infect the mosquito vector were not documented in the African apes [107]. Perhaps an extreme case, *Plasmodium* species in African Apes have generated an unusual situation where taxa with complete genomes may not have been “formally described.” Thus, primate malaria parasites highlight the problems describing and delimiting *Plasmodium* species.

Although using molecular data in the absence of morphology remains a contentious issue [85, 107], primate malarias have shown that single-gene criteria can first approximate species, particularly if multiple detections indicate active transmission and the data is of good quality.

Early molecular phylogenetic studies showed that the genus *Plasmodium* is not a monophyletic [36], an observation that has been confirmed ever since [25, 38, 39, 45, 108, 109]. Comparisons across phylogenetic analyses are not simply because of differences in taxon sampling and the loci used. Nevertheless, all molecular phylogenies show that *Plasmodium* is not monophyletic [25, 36, 38, 39, 45, 108, 109]. Indeed, the *Plasmodium* clade includes other genera found in mammals, such as *Polychromophilus* (Chiropteran), *Nycteria* (Chiropteran), and *Hepatocystis* (Chiroptera and Primates). It is worth noticing that, historically, the adoption of the genus *Plasmodium* was pragmatic [3, 4].

A critical early observation was that *P. falciparum* and *P. reichenowi* shared several features with avian parasite species, setting them apart from the other species infecting humans [3, 110–112]. Such discrepancies led to several proposed genera [3]. After an exhaustive review of the evidence, it was recommended that all human malaria parasites belong to the genus *Plasmodium*. The type species was settled in *P. malariae*. A separate subgenus, *Laverania*, was kept for the human parasite *P. falciparum* and *P. reichenowi* to set them apart from other primate malaria parasites that were classified under the subgenus, *Plasmodium*. Since taxonomy should integrate data into taxa, the generalization made 70 years ago was that the species that can produce malaria in humans belong to the genus *Plasmodium* (Opinion 283, IZCN cited in [3]). This recommendation was held even when many life-history traits that make a parasite a “*Plasmodium*” are not unique to the genus.

The elephant in the room is whether to revise *Plasmodium* and other Haemosporida genera based on molecular evidence. Unfortunately, given the practical importance of the current taxonomy, the problem is unlikely to be addressed soon. It is worth noticing that the two subgenera, *Plasmodium* and *Laverania*, seem to reflect those species’ biology. Furthermore, if additional evidence support that lemur parasites share a common ancestor with the non-*Laverania* primate malarias, this will lead to a monophyletic group of non-*Laverania* primate parasites that perhaps can be placed in the subgenus *Plasmodium*. Indeed, there was a rationale behind the subgenera while using the genus *Plasmodium* as an umbrella [4].

### *Laverania* and *Plasmodium* subgenus

Differences between these two clades of parasites fueled hypotheses about their distinct evolutionary histories. One that received attention was a “recent” origin of *P. falciparum* due to a host switch from an avian host, a hypothesis rooted in some particular interpretation of data, well before any formal phylogenetic analyses. It was argued that it explained the high virulence of *P. falciparum* compared to other human malarias [4, 56]. It lacked a precise timeframe of what was meant by “recent,” the term translated as “newer than the other human malaria parasites,” and the discussion seldom considered *P. reichenowi.*

However, virulence was not the more critical evidence. Avian malaria parasite species share morphological features with *P. falciparum* and *P. reichenowi,* such as falciform gametocytes [111, 112], placing them apart from *P. vivax* and *P. malariae*. Such resemblance drove the research agenda in avian malarias as they were noticed by Laveran (cited by [110]), leading to important discoveries such as mosquitoes as malaria vectors by Ross in 1898 using the avian parasite *P. relictum* as a model. Also, host switches were deemed common in avian malarias since morphologically indistinguishable species were found in hosts across avian families [4, 110], as modern studies have confirmed. Furthermore, some avian parasites were able to infect human erythrocytes experimentally [113]. Finally, early molecular evidence indicated that *P. falciparum* has similar A-T content in their genomes than avian and rodent malarias [114]. Thus, given the limited number of species with molecular data and the lack of a suitable outgroup, the tree topology of early phylogenetic studies was interpreted in such a context [88].

Although a spillover avian-origin for *P. falciparum* has been clearly rejected [2, 42–44, 61, 63, 89–91, 94, 97], the data separating *Laverania* from the *Plasmodium* subgenus remains. Indeed, as will be shown later, molecular traits are kept or lost in either one of these two subgenera compared to the common ancestor shared with avian parasites.

It is worth noticing that genome architecture features separate the subgenera *Laverania* and *Plasmodium*, including A + T content, patterns of codon usage, and the distribution of low-complexity regions [115–117]. Furthermore, among the best-known molecular adaptations separating *Laverania* from *Plasmodium* are gene families involved in antigenic variation [14, 49], such as the *var* gene family. Even gene families believed initially to be found across *Plasmodium* [49]*,* such as *Plasmodium*-interspersed repeat proteins (*pir*), do not have clear orthologs between the two clades of primate malarias [118]. However, here, the discussion will be limited to specific genes and loci without attempting an exhaustive review.

**Fig. 3:**
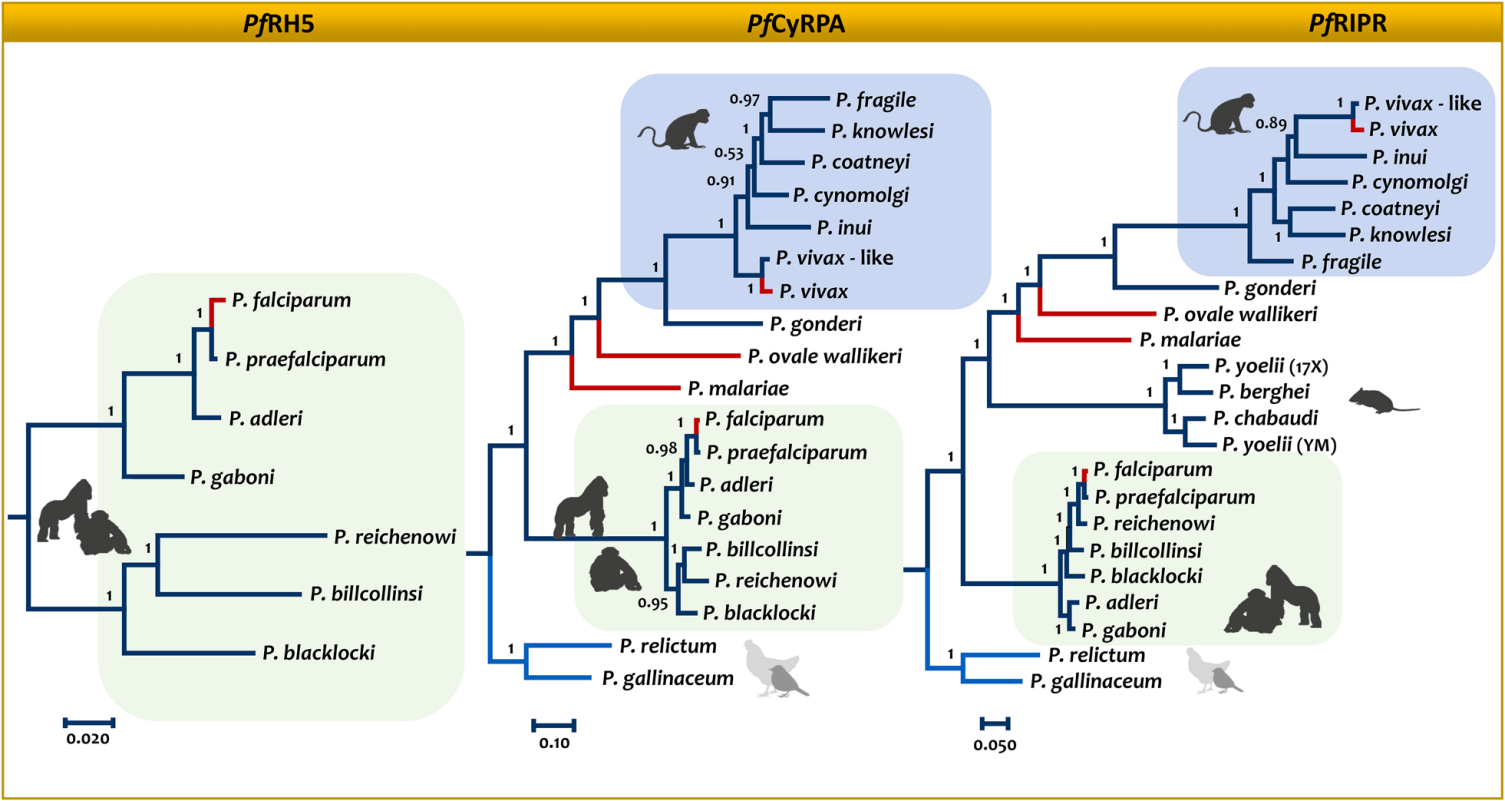
Evolution of the genes encoding the proteins in the RCR complex. Bayesian phylogenies for the proteins in the RCR complex were obtained using MrBayes v3.1.2. The two clades compared to assess differences in the strength of natural selection are indicated with different colors. In blue was the tested group corresponding with those closely related to *P. vivax;* the *Laverania* used as references are highlighted in green

Recently, the *Pf*RH5-*Pf*CyRPA-*Pf*Ripr (RCR) complex has been discovered in *P. falciparum;* it is essential in the invasion of the red blood cell and a target for the next generation of anti-*falciparum* vaccines [119, 120]. The *Pf*RH5-*Pf*CyRPA-*Pf*Ripr (RCR) complex is a protein trimer formed on the surface of the *P. falciparum* merozoite that binds to the host receptor basigin in the erythrocyte. The cell biology aspects of the complex have been revised elsewhere [119–121]. Here, what matters is that orthologs of *Pf*RH5 are found only in *Laverania,* but orthologs of *Pf*CyRPA and *Pf*Ripr are present in all primate malarias [121]. Figure 3 shows Bayesian phylogenetic analyses of the three genes encoding the proteins of the complex.

The *PfRipr* and *PfCyRPA* orthologs are essential in *P. knowlesi*, but they do not form a complex with each other [121]. Thus, the limited experimental evidence indicates that *cyrpa* and *ripr* orthologs have different functions in the two subgenera of primate malaria parasites. A simple and perhaps crude test of such functional differences can be exploring whether *Laverania* and the Asian species of the vivax-clade, where *P. knowlesi* and *P. vivax* are, have different selective regimens. In particular, by using phylogenetic-codon-based tests such as RELAX [122], changes in the selective regimens may indicate differences in function between the two clades. Using the vivax-clade as a test against the *Laverania* as a reference, this approach found evidence consistent with changes in how natural selection operates between the two groups. In particular, the vivax-clade shows relaxation for *cyrpa* gene when compared to *P. falciparum* (k = 0.62, *p* = 0.01). Relaxation in the strength of selection indicates a reduction in the intensity of natural selection in the test clade when compared against the reference, which may indicate a change in function. *Ripr* orthologs, on the other hand, show intensification in *P. vivax* when compared to *P. falciparum* (k = 14.81, p < 0.001). Notice that the reciprocal tests, *Laverania* as clade being tested and the vivax-clade as a reference, lead to the same results with different signs, so it is not worth commenting on them.

There are other examples among the proteins involved in the invasion of the red blood cell by merozoites, particularly the GPI-anchored proteins, that are known to be essential. Several genes have studied the issues regarding how natural selection may operate [85, 103, 123–125]. Here, the genes encoding the merozoite surface proteins 1 (*msp*1) and 2 (*msp*2) will be discussed as they have been the focus of studies for decades.

**Fig. 4:**
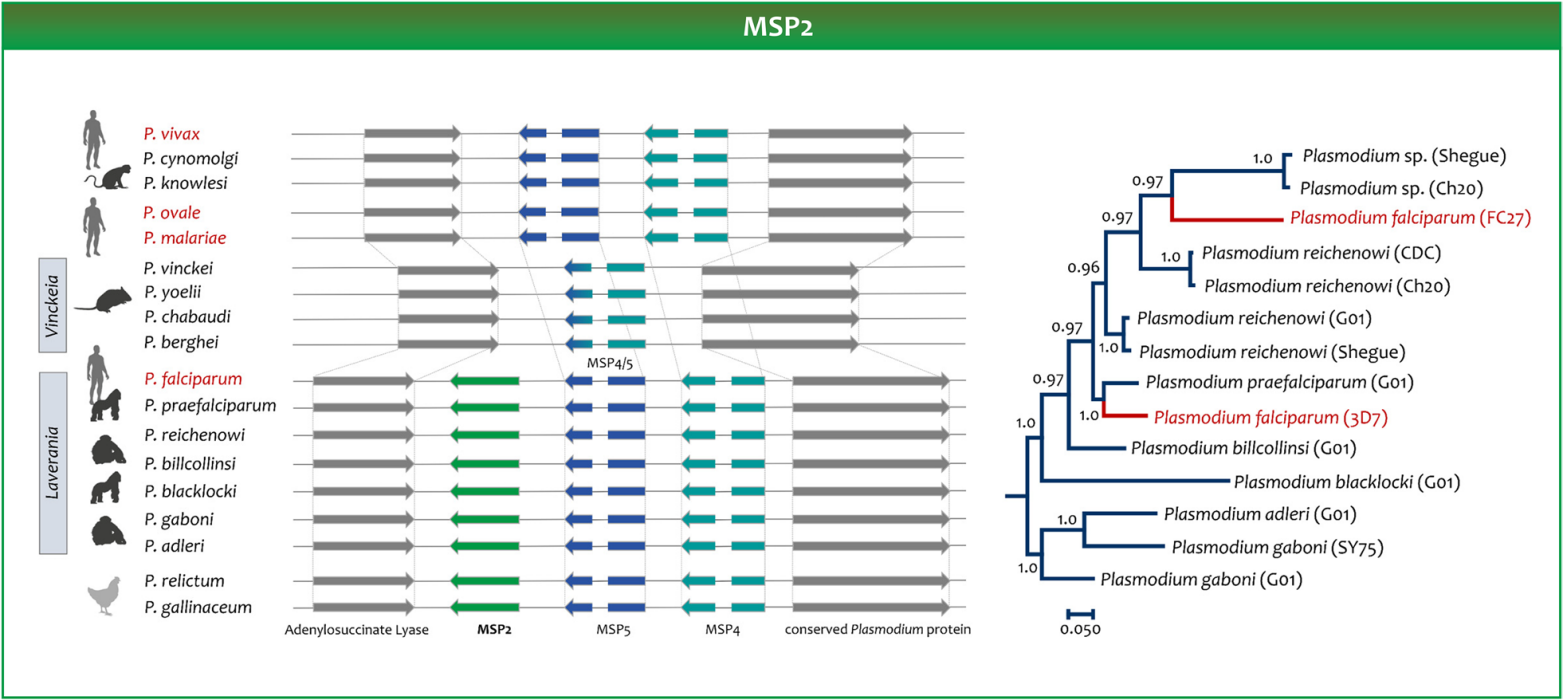
Evolution of the gene encoding the merozoite surface protein 2. A synteny map of the *msp2* region is depicted. A *msp2* ortholog is found in the avian parasites’ genomes. A Bayesian phylogeny obtained using MrBayes v3.1.2 on orthologous genes is provided. The branches with the two allelic forms in *P. falciparum* are colored in red

The gene encoding *msp*1 has a paralog in *P. vivax* (*Pvmsp1p*) that is highly conserved worldwide among populations of these parasites. The primary structure of *Pvmsp1p* protein contains a putative GPI anchor attachment signal and double epidermal growth factor (EGF)-like domains at the C terminus. This paralog is found in all the parasites in the subgenus *Plasmodium* and the two avian parasites included as outgroup (Fig. 4), but it was lost in the *Laverania* subgenus. Evidence indicates that this paralog may be part of a Duffy-independent pathway in *P. vivax* [126], so it may be an important mechanism to invade the red blood cell, as the conservation in multiple species indicates. In contrast, the gene encoding *msp1* is found in all *Plasmodium* species [103, 123, 124]. The *msp1* in *P. falciparum* is part of protein complexes with overlapping functions while interacting with human erythrocytes [127]. However, proteins that are part of those complexes in *P. falciparum,* such as *Pfmsp6* and *Pfmsp3*, only have orthologous in *Laverania*. It is worth notice what is called “*msp3*” in the vivax-clade are not orthologous with the genes with the same name in *Laverania* [128]. Thus, the lack of orthologous proteins that form such complexes is indicative of the differences between the two subgenera, even around essential functional proteins.

**Fig. 5:**
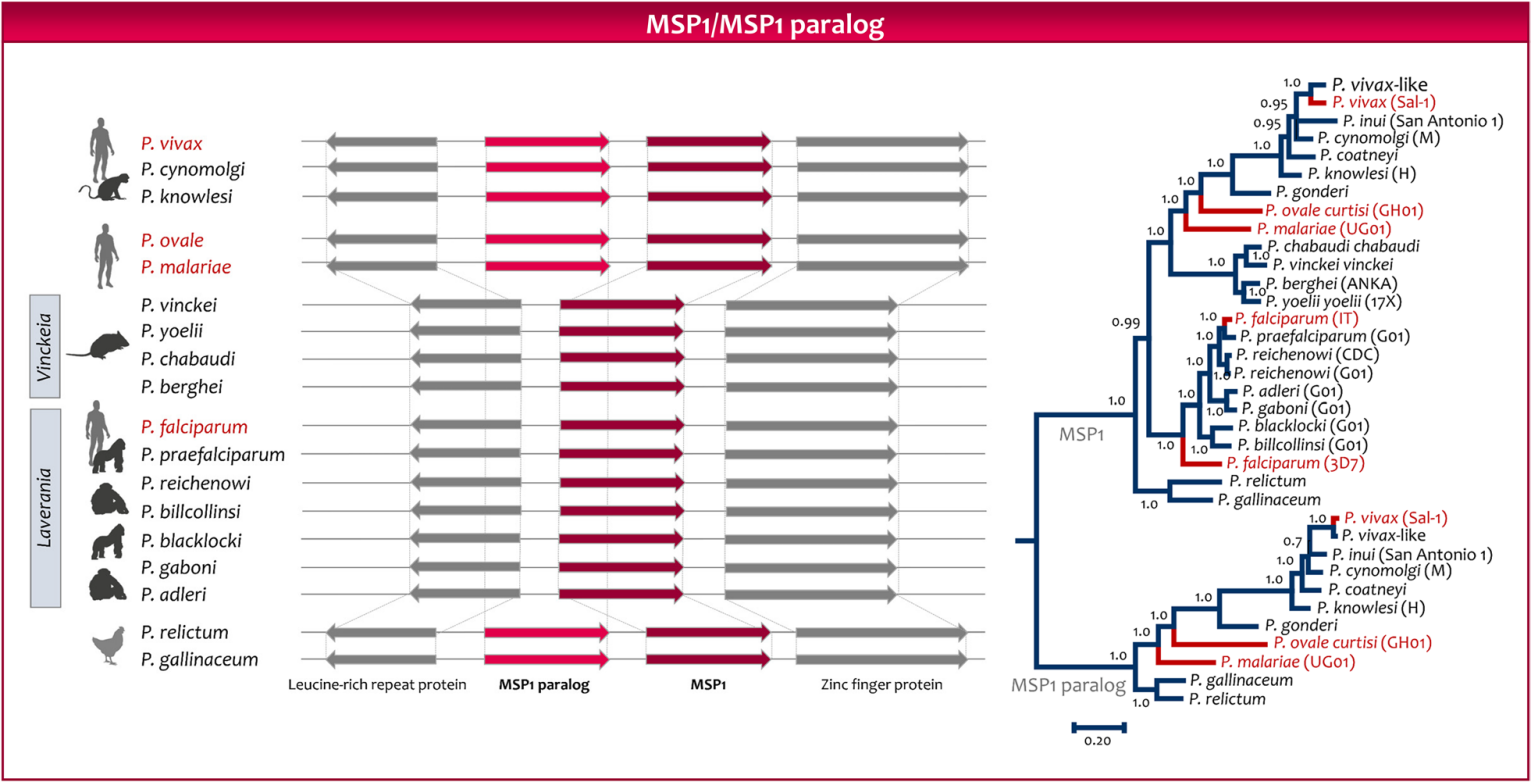
Evolution of the genes encoding the merozoite surface protein 1 and its paralog. A synteny map of the *msp1* region is depicted. Notice that an ortholog of the *msp1* paralog originally described in *P. vivax* is found in the genomes of avian parasites. A Bayesian phylogeny on orthologous genes obtained using MrBayes v3.1.2 is provided. The branches with the two *msp1* allelic forms in *P. falciparum* are colored in red. All the lineages leading to extant human malarias are indicated in red

The merozoite surface protein 2 (*msp2*) is an abundant GPI-anchored protein of *P. falciparum* that is expressed in the merozoite [123]. The *msp2* is an intrinsically disordered protein due to its variable central region. Indeed, the *msp2* polymorphism is still being used to characterize *P. falciparum* populations in molecular epidemiologic investigations [129]. Nevertheless, the N-terminal and C-terminal regions are highly conserved. All *msp2* alleles belong to either one of two allelic families, 3D7 and FC27, distinguished by their central variable regions [85]. These two allelic forms appear separated in the *msp2* Bayesian phylogeny depicted in Fig. 5. The gene encoding *msp2* is considered a *Laverania* specific gene since orthologs have not been found in other primate malarias [85]. Figure 5 shows the synteny of the orthologous genes encoding *msp2*; as expected, when only studying the *Plasmodium* in mammals, this gene can only be found in the *Laverania* clade. However, an ortholog encoding *msp2* is observed in the avian genomes. The implications are that, unlike the case of the *msp1* paralog, the *Laverania* clade kept *msp2* but that it was lost in the other *Plasmodium* infecting mammals.

The differences between the subgenera are not limited to blood stages. Recently, differences between the two subgenera have been made evident in the genes encoding chitinases. These genes are critical in releasing the ookinete in the vector, and this is perhaps the first documented difference in such a stage of the parasite life cycle [130]. There are differences in the chitinases between the vivax-clade and the *Laverania*. Species having two forms of chitinase seem to be an ancestral trait shared with the avian parasites. One form is preserved in *Laverania*, while the other is in *Plasmodium* subgenus and rodent malarias. However*, Plasmodium ovale* s.l. (both species) keeps the two forms of chitinases like in the avian malarias. Furthermore, *P. malariae* has one functional chitinase and a pseudogene [130]. Overall, this pattern indicates that losing one of the chitinases in *P. vivax* and related species is a relatively recent event in the evolution of the clade that includes all non-*Laverania* parasites [130].

### Molecular clock: timing the origin of the primate malaria clades

Considering the differences between the two *Plasmodium* clades in primates, when did they diverge? Numerous studies on time inferences have focused on *P. vivax* and *P. falciparum* [40, 97, 131–138]. An average mutation rate is usually estimated or assumed [97, 132, 134–137]. Such mutation rates are then used to infer the demographic histories of *P. vivax* or *P. falciparum* but do not address the genus’ origin. Importantly, those studies show a broad range of possible scenarios, most of which pointed to an expansion of those parasites following the human populations.

Perhaps a study that defers from the others used a tip-dating-based approach on an ancient genome of *P. vivax* [138]*,* a commonly used method in viruses. An assumption is that the time of collection informs about the mutation rate, so population structures before the sampling of the sequences are not supposed to introduce a bias. In this case, an isolate collected in Europe between 1942 and 1944 informs about the divergence concerning those in the Americas. The authors found a significant correlation between time of collection and divergence between isolates; however, it seems explained basically by two points, the ancient DNA isolate and the samples from North Korea. Only a few of the available isolates from the Americas were considered, and the possibility that the isolate collected in Europe was an introduction of *P. vivax* lineage from the Americas into Europe was not discussed. Regardless of this, it is an approach that seems interesting to explore and discuss further because it is the only one that may place *P. vivax* populations expanding in historical times [48, 132, 134, 135].

In contrast to the studies discussed above, there are few studies on the origin and radiation of the primate malaria parasites. Like when inferring demographic histories, various assumptions and data lead to different predictions. The first set of assumptions is calibrating the clock via time constraints (calibration points or time references). Primary calibration constraints, or direct evidence of a given event used as a reference, are independently provided from fossil data and known biogeographic events in the extant taxa [139]. There is almost no evidence of malarial parasites in the available fossil record, so host data is often used to inform the models [25, 131]. Those are secondary calibrations as they provide indirect evidence involving additional assumptions. By their very nature, those are problematic, but there seems no way of avoiding them. Thus, calibration constraints should be carefully described to be tested by others [25, 140], understanding that such analyses simply make some scenarios more parsimonious than others.

The second set of assumptions involves how to model the rate of evolution, constant or heterogeneous. It has been long known that a constant rate of evolution model (strict molecular clock) is rejected when including more distant species [139, 141]. Nevertheless, variations of a single rate of evolution or some form of constant rate have been widely used in *Plasmodium*.

Perhaps the first modern molecular timing analyses were carried out assuming a strict clock model on the mitochondrial genome. The assumption of constant rate was statistically rejected [142]. As an ad hoc approach, a rate was estimated on a subset of species where the strict clock was not rejected. Then the rate was extrapolated on the other species [142]. The origin of the genus was estimated to be 22.2–41.6 Ma (million years ago), and the time to the common ancestor between *Plasmodium* in primates and rodents was estimated to be 18.3–34.7 Ma. The origin of the Catarrhini primate malarias was estimated to be 25.7 Ma [23.1–28.3] under one of the scenarios. This estimate overlaps with fossils that place such events between 24 and 34 Ma [143]. The time estimates were younger than their hosts for all the other clades, which could be interpreted as host-switches. However, in addition to the assumption of constant rate, the sampling of taxa could have also been a factor since most species were primate malaria parasites.

A modality of a constant rate of evolution estimated the time of origins of a few *Plasmodium* species by using genomic level data of coding protein genes [144]. In contrast to the previous study [142], this approach estimated substantially older times for the origin of *Plasmodium* in primates and rodents. The method proposed calculated relative times against a reference divergence between a pair of species. *Plasmodium vivax* and *P. knowlesi* were used as a reference in the original study, but changing the pair of species used was not explored. Nevertheless, the method assumes a constant rate of evolution for each ortholog group of single-copy protein genes across species, not a single rate for all proteins. Under these assumptions, the split between the human parasite *P. falciparum* and the rodent parasite *Plasmodium yoelii* was 6.1 times older than the split of the chimpanzee parasite *P. reichenowi* from the human parasite *P. falciparum* [144]. Thus, estimates depend on how to convert such relative times.

Although having a single and constant mutation rate seems desirable, it is not a robust approach [139, 141, 145]. Furthermore, if such rates are estimated using ad hoc methods, the analyses are difficult to replicate. Thus, mainstream molecular dating approaches are desirable because different scenarios, methods, and assumptions can be compared.

Overall, given the data, Bayesian methods incorporate prior knowledge to estimate a posterior distribution in times and a phylogeny [141]. Priors include the values used as calibrations and modeling the rate heterogeneity and substitution models. Multiple calibrations do not imply that they all have the same effect [146], so it is important to explore different scenarios. Bayesian methods also model rate heterogeneity; the two basic models assume autocorrelation or independence. In the autocorrelation model, evolutionary rates are correlated by descent because of similarities between ancestral and descendent species [139, 141]. The independent rate variation model, on the other hand, assumes that rates vary throughout the tree following a probability distribution but are not affected by ancestry [139, 141]. Those models of evolutionary rate variation could affect estimates in particular data sets and should be compared whenever possible.

It is also noted that adding species without calibration likely increases the variance in time estimates because they may add heterogeneity in the rates [139, 141]. On the other hand, more loci may be beneficial if they have congruent phylogenetic signals, are not saturated, share patterns of rate variation across lineages, and do not require different substitution models (e.g., similar GC content). The latest is challenging to accomplish in *Plasmodium* genus because of the differences in GC content [115], but the mitochondria and apicoplast genomes offer an alternative [39, 147]. Of the two, the mitochondria have been widely used in such molecular clock studies.

A first attempt to explore alternative scenarios (more than a single evolutionary rate) used two different calibrations within primates [25]. This study also compared two Bayesian methods, one that assumed autocorrelation and the other independent rates. The estimates with the autocorrelation model yielded slightly older times than the independent rate models. However, given the phylogeny's limited species sampling, the credibility intervals of the estimated times between the two methods overlapped. The study found that there was some overlap with the estimates of Hayakawa et al. [142]. The novelty of this study was that it used scenarios that could explain the origin of the parasite in lemurs to validate the time estimates obtained by using separated calibrations [25]. These scenarios were further investigated, considering different calibrations and assumptions in rodent malarias [148], producing similar results.

An expanded phylogeny incorporating 102 mitochondrial genomes was constructed, including data from avian species parasites of the genera *Leucocytozoon*, *Haemoproteus*, and *Plasmodium* [39, 149]. It was found that different models of evolutionary rate variation across lineages, independent or autocorrelated, affect time estimates, particularly in avian clades [39]. Overall, the study estimated that the time of origin of *Plasmodium* in primates and rodents was approximately 45 Ma. Unlike early studies, the *P. reichenowi* and *P. falciparum* split were not included as calibration, but it is estimated around 6 Ma (4–8.4 Ma), coinciding with the *Homo-Pan* common ancestor. These time estimates remind consistent even when introducing a genus sharing a recent common ancestor with *Plasmodium* as is *Haemocystidium* in reptiles [149].

A limitation in all mitochondrial studies is the lack of important gorilla lineages. The sequences available from this organelle from several ape malarias are too short, so their inclusion increases the uncertainty in time estimates [25]. The additional problem is that the mitochondrial genome, as described earlier, undergoes different selective regimes correlated with the families of vector species [39]. This concern is mitigated because all mammalian malarias are transmitted by *Anopheles* mosquitoes, and all calibrations used are within primate malarias. Still, it is important to include other loci as they become available.

Although these studies used secondary calibrations [25, 39, 149], it was found that the calibration points were internally consistent. That is, removing one calibration does not lead to incongruent time estimates. The times’ estimates seem to be consistent even when including a calibration out of the primate-rodent malaria clade, as is the origin of the *Plasmodium* found in ungulates [39, 149]. Therefore, given the data, these time priors provide a plausible framework for the timing primate malarias. Nevertheless, the best way to move the field forward is to seek additional calibration constraints, increase the number of loci and improve the sampling of taxa.

What has been learned about the time of the radiation of primate malarias? It seems that the two clades of primate malaria parasites, those under the subgenera *Plasmodium* and *Laverania*, diverged early on, with the origin of Catarrhini primates [143]. Considering the evolutionary histories of their extant hosts, the origins of these two clades likely took place in Africa. How this relates to the other none-*Plasmodium* genera that evolved intertwined with these two primate clades is an issue that needs to be investigated.

## Conclusions

Although a challenge for control and elimination, the biodiversity of malarial parasites in primates makes this a unique model for those interested in the evolutionary biology of parasites and the use of comparative approaches. Indeed, to those interested in particular molecular adaptations or understanding metabolic pathways, comparing distinct *Plasmodium* species that still have common (and sometimes analogous) traits allows placing discoveries in a general evolutionary context.

The evidence indicates that host-switches are common; however, there seems to be some level of host-specificity that, together with biogeographical processes, can explain the observed diversity of primate malarias. The two clades that include primate malarias diverged early in the evolution of their hosts. Such early divergence translates into traits comprising everything from single genes to gene families. Even when using subgenera is not practical in several contexts, they remind us of the differences between the primate malaria clades in a taxonomical framework that is unlikely to be changed soon.

The molecular evidence supports an African origin for the two clades of primate malaria parasites with a species radiation in Southeast Asia from lineages that originated in Africa, likely following their hosts’ populations expansions. Such cladogenesis is consistent with adaptive radiation as several phenotypes emerged de novo.

Timing the evolution of primate malarias requires additional data; however, the available estimates offer a framework consistent with the phylogeny and biogeography of these parasites’ hosts. Although consistency does not make these times estimates correct, it is a hypothesis to be tested as more species and data are included.

A pending matter in primate malaria biodiversity is characterizing those parasites found in lemurs and gibbons. Understanding how the molecular adaptations found in the primate malaria clades originated is of critical importance. Answering such questions will require scaling up comparative genomic studies to include more malaria parasite species from nonhuman primates and other Haemosporida genera that share a common history with what is known as the genus *Plasmodium* s.l. In conclusion, paraphrasing Dr. William (“Bill”) Collins, an authority in primate malaria parasites and one of the authors of the primate malarias book [3], “we learn from all the parasites.”

## Data Availability

Not applicable.

## Abbreviations

18s SSU rRNA: Structural RNA for the small subunit of ribosomes
CSP: Circumsporozoite protein
*cytb*: Mitochondrial cytochrome b gene
IZCN: The International Code of Zoological Nomenclature
Ma: Million years ago
*msp1*: Merozoite surface protein 1
*msp2*: Merozoite surface protein 2
*msp3*: Merozoite surface protein 3
*Pf*RH5: Reticulocyte Binding Protein Homologue 5 in *P. falciparum*
*Pf*CyRPA: Cystine-Rich Protective Antigen in *P. falciparum*
*Pf*Ripr: Merozoite Rh5 interacting protein in *P. falciparum*
*Pfmsp6*: Merozoite surface protein 6 in *P. falciparum*
s.l.: Sensu lato
